# Vitamin D Related Gene Polymorphisms and Cholesterol Levels in a Mediterranean Population

**DOI:** 10.3390/jcdd9040102

**Published:** 2022-03-27

**Authors:** Hana M. A. Fakhoury, Said El Shamieh, Amru Rifai, Hani Tamim, Rajaa Fakhoury

**Affiliations:** 1 Department of Biochemistry and Molecular Biology, College of Medicine, Alfaisal University, P.O. Box 50927, Riyadh 11533, Saudi Arabia; hana.fakhoury@gmail.com (H.M.A.F.); aalrifai@alfaisal.edu (A.R.); htamim@aub.edu.lb (H.T.); 2 Department of Medical Laboratory Technology, Faculty of Health Sciences, Beirut Arab University, Beirut 11-5020, Lebanon; s.elshamieh@bau.edu.lb; 3 Department of Internal Medicine, American University of Beirut Medical Center, Beirut 11-0236, Lebanon

**Keywords:** vitamin D, CYP2R1, rs10741657, LDL cholesterol, HDL cholesterol, single nucleotide polymorphisms, association analysis

## Abstract

In addition to its role in bone health, vitamin D (VitD) has been implicated in several pathological conditions. Specifically, VitD deficiency has been linked to an increased risk of dyslipidemia. Atherogenic dyslipidemia is characterized by increased low-density lipoprotein-cholesterol (LDL-C) and decreased high-density lipoprotein-cholesterol (HDL-C). In this study, we examined the association of six single nucleotide polymorphisms (SNPs) in VitD-related genes with VitD and lipid levels, in a cohort of 460 Lebanese participants free from chronic diseases. Our results showed no association of the examined SNPs with VitD concentrations. However, the presence of the minor allele in rs10741657G>A of *CYP2R1* was associated with increased levels in LDL-C (β = 4.95, *p* = 0.04)] and decreased levels in HDL-C (β = −1.76, *p* = 0.007)]. Interestingly, rs10741657G>A interacted with gender to increase LDL-C levels in females (β = 6.73 and *p* = 0.03) and decrease HDL-C levels in males HDL-C (β = −1.09, *p* = 0.009). In conclusion, our results suggest that rs10741657 G>A in *CYP2R1* is associated with circulating LDL-C and HDL-C levels in a Lebanese cohort. Although this association was gender-specific, where rs10741657G>A was associated with increased LDL in females and decreased HDL in males, the presence of the minor allele A was associated with increased cardiovascular risk in both genders. These findings need to be validated in a larger population. Further investigations are warranted to elucidate the molecular mechanism of VitD polymorphism and dyslipidemia.

## 1. Introduction

In addition to its major role in skeletal health, vitamin D (VitD) has been implicated in several pathological conditions [1,2]. Indeed, VitD deficiency has been suggested to contribute to the pathogenesis of many disorders, including autoimmune, infectious, and cardiovascular diseases [1,2,3,4,5]. Noticeably, VitD deficiency has been linked to an increased risk for dyslipidemia [6,7,8]. Atherogenic dyslipidemia is characterized by increased low-density lipoprotein-cholesterol (LDL-C) and decreased high-density lipoprotein-cholesterol (HDL-C).

Exposure to sunlight converts 7-dehydrocholesterol (7-DHC) in the skin to vitamin D [9]. Vitamin D binding protein [10] (VDBP) transports VitD to the liver, where it is hydroxylated by the liver enzyme 25-hydroxylase (CYP2R1) to its main circulating form 25-hydroxyvitamin D [25(OH)D] [11,12]. It has been well established that circulating levels of 25(OH)D are influenced by a multitude of factors, including age, gender, diet, supplementation, sun exposure, latitude, and race [13,14,15,16,17,18,19]. More recently, significant attention has been given to the genetic determinants of VitD status. Indeed, genome-wide association studies [20,21,22] have demonstrated that polymorphic loci in or near genes encoding for VitD related proteins are associated with 25-(OH) D concentrations. These loci include the *CYP2R1* gene, the *GC* gene encoding for VDBP, and the *NADSYN1/DHCR7* gene region. However, studies on these genetic associations remain scarce in the Mediterranean region. Based on the above, we aimed to study the effect of six single nucleotide polymorphisms (SNPs) in *CYP2R1*, *GC*, and *NADSYN1/DHCR7* on VitD concentrations in 460 Lebanese individuals free of chronic disease. We also investigated the association of these SNPs with levels of low-density lipoprotein-cholesterol (LDL-C) and high-density lipoprotein-cholesterol (HDL-C).

## 2. Materials and Methods

### 2.1. Study Participants

This cross-sectional study was conducted on 460 unrelated Lebanese individuals. Participants were recruited from a major tertiary care hospital in Lebanon between 2015 and 2016. Individuals with a diagnosis of cardiovascular disease or cancer were excluded from the study.

### 2.2. Ethical Statement and Recruitment

This study adhered to the latest version of the Declaration of Helsinki for Ethical Principles for Medical Research Involving Human Subjects during recruitment and data collection procedures. The Institutional Review Board of the Beirut Arab University approved the study (2019-H-0091-HS-R-0360). Informed consent was obtained from all participants.

### 2.3. Clinical, Biological, and Genetic Data

A questionnaire was used to assess the socio-demographic data. Measurements of serum concentrations of 25(OH)D and lipid profile were performed using commercial kits (Roche Diagnostics, Basel, Switzerland).

Peripheral blood samples drawn in EDTA tubes were used to extract the genomic DNA (QIAamp DNA blood mini kit, Qiagen, Hilden, Germany). All samples were genotyped using a Kompetitive allele-specific PCR as described previously [23] for rs10741657G>A in *CYP2R1*, rs12785878 T>G, rs4423214 T>C, rs4944062T>G in *NADSYN1/DHCR7*, and rs4588 C>A, rs2282679 A>C in GC.

### 2.4. Statistical Analysis

SPSS Statistics software was used to perform the statistical analysis (Version 22.0, Armonk, NY, USA). Scale and categorical variables were presented as mean ± standard deviations and numbers followed by percentages, respectively. A Chi-square test was used to determine if the genotypes were in Hardy–Weinberg equilibrium. Multivariate linear regression analysis (adjusted for physical activity, body mass index, smoking, age, and gender) was used to study the association between rs10741657G>A in *CYP2R1* and the lipid profile. An interaction between rs10741657G>A x gender on lipid profile was also assessed. The additive model was tested, and the significance level was set at *p* < 0.05.

## 3. Results

The demographic, clinical, and genetic characteristics of our participants are shown in Table 1. There was no significant difference in the age of males versus females (Table 1). Men had significantly higher BMI than women, while women had higher levels of total cholesterol, HDL-C, LDL-C, and circulating 25(OH)D than men (Table 1). The genotypic distribution of the studied SNPs was in agreement with the Hardy-Weinberg equilibrium (Table 1). No significant difference was found in the distribution of the 6 SNPs between males and females (Table 1, *p* > 0.05).

Among the tested SNPs, only the A allele of rs10741657G>A in *CYP2R1* was associated with increased levels of LDL-C (β = 4.95, *p* = 0.04, Table 2), and decreased levels of HDL-C (β = −1.76, *p* = 0.007, Table 2). As expected, age, gender, smoking, and physical activity showed significant associations (*p* < 0.05, Table 2).

In order to examine whether rs10741657G>A in *CYP2R1* and gender may indirectly influence lipid levels, we tested their interaction on LDL-C, HDL-C using multivariate linear regression models (Table 3 and Table 4). Interestingly, rs10741657A was associated with an increased risk of high LDL-C in females (β = 6.73 and *p* = 0.03) but not in males (Table 3). Age was significantly associated with LDL-C levels in both males (*p* = 0.001) and females (*p* = 0.01). Alcohol consumption was associated with decreased LDL-C in males only (*p* = 0.002). Physical activity was associated with decreased LDL-C in females only. BMI was conversely associated with LDL-C in females (*p* < 0.0001) (Table 3).

Interestingly, when stratified according to gender, rs10741657A was associated with decreased levels of HDL-C in males (β = −1.09, *p* = 0.009) (Table 4). On the other hand, age was associated with increased HDL-C in males (*p* = 0.004) and decreased HLD-C levels in females (*p* = 0.001). Smoking was associated with increased HDL-C levels in both males (*p* = 0.001) and in females (*p* < 0.0001). BMI was associated with decreased HDL-C in both males (*p* = 0.02) and females (*p* = 0.04) (Table 4).

## 4. Discussion

Our results showed no association of VitD concentrations with the examined SNPs in the studied Lebanese population. However, we found that rs10741657A in *CYP2R1* was associated with increased levels of LDL-C and decreased levels of HDL-C. The stratification according to gender revealed that rs10741657A interacted with gender to increase LDL-C levels in females and decrease HDL-C levels in males. Although this association may appear to be gender-specific, the outcome is comparable since dyslipidemia is characterized by increased low-density lipoprotein-cholesterol (LDL-C) and decreased high-density lipoprotein-cholesterol (HDL-C). This finding is comparable to a Finnish study, where another SNP of *CYP2R1*, rs12794714, was associated with LDL-C [24].

It is worth noting that this study is limited by its small sample size; replication studies in a larger cohort are warranted. Another limitation is the lack of information on the diet of the participants and other confounding factors such as the season of sample collection.

As mentioned earlier, our analysis did not show an association between VitD concentrations and the examined SNPs. This finding is in line with a study on a British population [25] where none of the studied SNPs was associated with VitD concentrations. Although Arabi et al. demonstrated a gender-specific association between rs10741657G>A in *CYP2R1* and the VitD status of a Lebanese cohort [26], this discrepancy might be due to the small sample size and the sampling conditions where the level of vitamin D is influenced by seasonal variation [27,28].

Herein, we found that rs10741657G>A in the gene involved in the hydroxylation activity of VitD affects the level of cholesterol by increasing LDL-C and decreasing HDL-C. In the interpretation of these results, it is worth noting that VitD and cholesterol metabolism are related since both molecules share a common metabolic substrate, 7-DHC. Indeed, in the final step of cholesterol biosynthesis, 7-DHC is converted to cholesterol by 7-DHCR; alternatively, 7-DHC could be converted to VitD in the skin upon sun exposure.

In conclusion, our results suggest that rs10741657A in *CYP2R1* is associated with circulating LDL-C and HDL-C levels. Although the association appears to be gender-specific, where rs10741657G>A was associated with increased LDL in females, and decreased HDL in males, the presence of the minor allele was associated with increased cardiovascular risk in both genders. These findings need to be validated in a larger population. Further investigations are needed to elucidate the association of VitD polymorphism with dyslipidemia. Understanding this relationship may allow the development of dietary interventions that could reduce the risk of developing dyslipidemia.

## Figures and Tables

**Table 1:** Demographic, clinical, and genetic characteristics of the study participants.

	Total	Female Mean (SD)	Male Mean (SD)	*p*
	N = 460	N = 292	N = 168	
Age	40.60 (14.16)	39.80 (12.85)	41.98 (16.13)	0.1118
BMI (Kg/m^2^)	25.71 (4.98)	24.53 (4.56)	27.78 (5.02)	<0.001
Total cholesterol (mg/dl)	181.41 (40.94)	185.86 (40.68)	173.69 (40.36)	0.0021
HDL-C (mg/dl)	45.54 (14.61)	48.46 (14.71)	40.45 (12.97)	<0.001
LDL-C (mg/dl)	117.39 (33.52)	120.28 (34.10)	112.37 (31.99)	0.0147
25(OH)D (ng/mL)	24.53 (13.81)	25.62 (17.56)	22.64 (9.26)	0.0253
Genotype				
rs10741657G>A in *CYP2R1*				
GG	206 (50.4)	129 (50.4)	77 (50.3)	0.71
AG	165 (40.3)	101 (39.5)	64 (41.8)	
AA	38 (9.3)	26 (10.3)	12 (7.8)	
rs12785878T>G in *NADSYN1/DHCR7*				
TT	116 (27.0)	73 (26.8)	43 (27.4)	0.77
GT	211 (49.2)	137 (50.4)	74 (47.1)	
GG	102 (23.8)	62 (22.8)	40 (25.5)	
rs4588C>A in *GC*				
CC	267 (62.2)	166 (61.3)	101 (63.9)	0.48
AC	140 (32.6)	93 (34.3)	47 (29.7)	
AA	22 (5.1)	12 (4.4)	10 (6.3)	
rs2282679A>C in *GC*				
AA	272 (62.5)	172 (62.1)	100 (63.3)	0.35
CA	143 (32.9)	95 (34.3)	48 (30.4)	
CC	20 (4.6)	10 (3.6)	10 (6.3)	
rs4423214T>C in *NADSYN1/DHCR7*				
TT	114 (26.4)	73 (26.4)	41 (26.5)	0.74
CT	215 (49.8)	141 (50.9)	74 (47.7)	
CC	103 (23.8)	63 (22.7)	40 (25.8)	
rs4944062T>G in *NADSYN1/DHCR7*				
TT	115 (27.3)	74 (27.4)	41 (27.0)	0.54
GT	211 (50.0)	139 (51.5)	72 (47.4)	
GG	96 (22.7)	57 (21.1)	39 (25.7)	

Values were arithmetic mean ± standard deviation for scale variables. Categorical variables were shown as numbers and percentages. BMI: body mass index, HDL-C: High-density lipoprotein cholesterol, LDL-C: Low-density lipoprotein cholesterol, VitD: Vitamin D.

**Table 2:** Multivariate linear regression analysis with low- and high-density lipoproteins cholesterol.

	LDL-C	
	β (95%CI)	*p*-Value
rs10741657A	4.95 (0.20; 9.70)	0.04
Age	0.54 (0.32; 0.76)	<0.0001
Physical activity		
<1 week	Reference	
1 week	−1.39 (−1.06; −1.72)	<0.0001
Gender		
Female	Reference	
Male	−1.40 (−1.84; −1.96)	0.004
Smoking		
No	Reference	
Yes	−1.41 (−1.97; −1.86)	0.003
	HDL-C	
rs10741657A	−1.76 (−1.76; −1.77)	0.007
Gender		
Female	Reference	
Male	−1.37 (−1.24; −1.50)	<0.0001
Smoking		
No	Reference	
Yes	3.57 (2.10; 5.06)	<0.0001
BMI	−1.43 (−1.71; −1.15)	0.003

β: regression coefficient, LDL-C: low-density lipoprotein cholesterol, HDL-C: high-density lipoproteins cholesterol. R^2^ for LDL-C model is: 0.109, R^2^ for HDL-C model is: 0.113.

**Table 3:** Interaction analysis between rs10741657 and gender on low-density lipoprotein cholesterol, using stepwise regression model.

	LDL-C	
	β (95%CI)	*p*-Value
Male		
rs10741657	1.03 (−1.89; 7.96)	0.77
Age	0.47 (0.19; 0.75)	0.001
Alcohol		
No	Reference	
Yes	−1.25 (−1.80; −1.69)	0.002
Female		
rs10741657	6.73 (0.61; 12.86)	0.03
Age	0.41 (0.09; 0.73)	0.01
Physical activity		
<1 week	Reference	
1 week	−1.88 (−1.47; −1.29)	<0.0001
>1 week	−1.38 (−1.04; −1.73)	0.02
BMI	−1.75 (−1.66; −1.84)	<0.0001
rs10741657*gender interaction		0.005

Factors included in the model were age, gender, physical activity, alcohol consumption, and BMI. Variables significant at stepwise analysis were reported in the table. β: regression coefficient, LDL-C: low-density lipoprotein cholesterol, BMI: body mass index. R^2^ for male model is: 0.153, R^2^ for female model is: 0.141, R^2^ for interaction model is: 0.108.

**Table 4:** Interaction analysis between rs10741657 and gender on high-density lipoprotein cholesterol levels.

	HDL-C	
	β (95%CI)	*p*-Value
Male		
rs10741657	−1.09 (−1.15; −1.04)	0.009
Age	0.18 (0.06; 0.30)	0.004
smoking		
No	Reference	
Yes	4.04 (1.65; 6.44)	0.001
BMI	−1.48 (−1.89; −1.08)	0.02
Female		
rs10741657	−1.77 (−1.32; 0.77)	0.17
Age	−1.23 (−1.36; −1.10)	0.001
smoking		
No	Reference	
Yes	3.79 (1.89; 5.69)	<0.0001
BMI	−1.39 (−1.77; −1.01)	0.04
rs10741657A*gender interaction		<0.0001

Factors included in the model were age, gender, physical activity, alcohol consumption, and BMI. Variables significant at stepwise analysis were reported in the table. β: regression coefficient, DL-C: high-density lipoproteins cholesterol, BMI: body mass index. R^2^ for interaction model is: 0.145, R^2^ for male model is: 0.192, R^2^ for female model is: 0.105.

## Data Availability

Please contact authors for data requests.
